# Novel Approach Combining Shallow Learning and Ensemble Learning for the Automated Detection of Swallowing Sounds in a Clinical Database

**DOI:** 10.3390/s24103057

**Published:** 2024-05-11

**Authors:** Satoru Kimura, Takahiro Emoto, Yoshitaka Suzuki, Mizuki Shinkai, Akari Shibagaki, Fumio Shichijo

**Affiliations:** 1 Division of Science and Technology, Graduate School of Sciences and Technology for Innovations, Tokushima University, Tokushima 770-8506, Japan; kimura.s0126@gmail.com; 2 Division of Science and Technology, Industrial and Social Science, Graduate School of Technology, Tokushima University, Tokushima 770-8506, Japan; 3 Department of Stomatognathic Function and Occlusal Reconstruction, Graduate School of Biomedical Sciences, Tokushima University, Tokushima 770-8504, Japan; yosuzuki@tokushima-u.ac.jp (Y.S.); c302151006@tokushima-u.ac.jp (M.S.); c302251023@tokushima-u.ac.jp (A.S.); 4 Department of Neurosurgery, Suzue Hospital, Tokushima 770-0028, Japan; shichijo_7@mac.com

**Keywords:** swallowing sound, automated detection

## Abstract

Cervical auscultation is a simple, noninvasive method for diagnosing dysphagia, although the reliability of the method largely depends on the subjectivity and experience of the evaluator. Recently developed methods for the automatic detection of swallowing sounds facilitate a rough automatic diagnosis of dysphagia, although a reliable method of detection specialized in the peculiar feature patterns of swallowing sounds in actual clinical conditions has not been established. We investigated a novel approach for automatically detecting swallowing sounds by a method wherein basic statistics and dynamic features were extracted based on acoustic features: Mel Frequency Cepstral Coefficients and Mel Frequency Magnitude Coefficients, and an ensemble learning model combining Support Vector Machine and Multi-Layer Perceptron were applied. The evaluation of the effectiveness of the proposed method, based on a swallowing-sounds database synchronized to a video fluorographic swallowing study compiled from 74 advanced-age patients with dysphagia, demonstrated an outstanding performance. It achieved an F1-micro average of approximately 0.92 and an accuracy of 95.20%. The method, proven effective in the current clinical recording database, suggests a significant advancement in the objectivity of cervical auscultation. However, validating its efficacy in other databases is crucial for confirming its broad applicability and potential impact.

## 1. Introduction

Dysphagia is a disease that is frequently caused by stroke; cerebrovascular, neurogenerative (e.g., Alzheimer’s disease or Parkinson’s disease), or neuromuscular disease; or aging [1,2,3,4], with a particularly high morbidity rate among older patients, and is characterized by symptoms such as choking or coughing fits when eating, or by saliva incorrectly entering the trachea or lungs. These symptoms, in turn, may cause aspiration pneumonia, which is a serious disease that is among the top causes of death worldwide [5,6,7]. Therefore, dysphagia warrants early diagnosis and proper rehabilitation. Current methods used to diagnose dysphagia include a video fluorographic swallowing study (VFSS) and a fiberoptic endoscopic examination of swallowing (FEES) [8,9,10,11,12,13,14].

Both VFSS and FEES are effective for diagnosing swallowing disorders, but each has its advantages and limitations. Cervical auscultation (CA) is a useful noninvasive method for assessing swallowing disorders; however, it has limitations as a diagnostic tool due to issues of subjectivity and accuracy [15,16]. According to a systematic review by Lagarde et al. [17], the diagnostic accuracy of CA varies widely, with sensitivity ranging from 23% to 95% and specificity from 50% to 74%. This wide range reflects the susceptibility of CA results to subjective interpretation, which raises questions about its reliability, especially when not combined with acoustic analysis or other sophisticated methods [17]. Furthermore, advancements in digital signal processing technology have improved the analysis of swallowing sounds obtained through CA. The data from swallowing sounds and vibrations are analyzed through specific digital processing steps, and the results are utilized to evaluate swallowing disorders. Such technology is expected to enhance the accuracy of CA signals and reduce subjectivity [18].

The evaluation of the deglutition function based on swallowing sounds is being investigated [19,20,21,22]. Rayneau et al. developed and evaluated the effectiveness of a smartphone application for automatically detecting and analyzing swallowing sounds in healthy individuals and patients with pharyngeal cancer when engaged in specific types of eating. Based on the results, swallowing sounds were detected with 90% or higher sensitivity in healthy persons when consuming all three food forms (mashed potatoes, water, and yogurt) that were tested; however, the application of the method in patients with cancer proved difficult [20]. Among various studies, the analysis of swallowing sounds using machine learning is a particularly promising approach. Frakking et al. used a Support Vector Machine (SVM) to identify swallowing sounds in healthy children and children with dysphagia and demonstrated 98% accuracy [21], whereas Sarraf Shirazi et al. used K-means clustering to detect aspiration, with 86.4% accuracy, from a sub-band frequency of 300 Hz or less [22].

Advances in machine learning confer the possibility of further improvement of accuracy when detecting swallowing sounds [22,23,24,25]. Khlaifi et al. combined the Mel Frequency Cepstral Coefficient (MFCC) and a Gaussian Mixture Model (GMM) to achieve an 84.57% recognition rate [23]. In a study by Kuramoto et al., a recording database of healthy persons and patients with dysphagia was used to apply a deep learning model wherein the swallowing sounds versus noise were distinguished with a high accuracy of 97.3%, and showed that deep learning can effectively support a diagnosis of dysphagia [24]. However, according to our findings, and focusing on the peculiar features of the patterns of swallowing sounds in real-world clinical conditions, research into their detection is lacking. Additionally, deep learning models require a large data set and advanced computational resources.

This study was conducted with the aim of enabling the automatic detection of swallowing sounds from a clinical recording database by using a novel approach and evaluating the performance thereof. 

## 2. Proposed Method

### 2.1. Recording Database 

**Figure 1:**
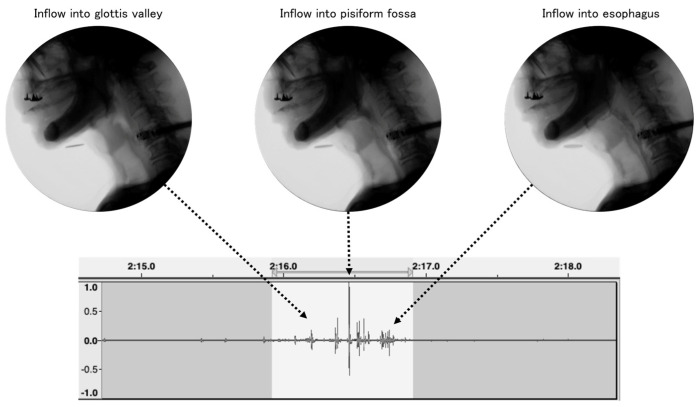
Example of swallowing sounds detected from video fluorographic swallowing study (VFSS).

Audio recordings were made of 74 patients having or suspected of having dysphagia who they consented to participate in this study. The participants ingested 2–3 g of gelatinous jelly; then, using a throat microphone (SH-12jk, NANZU ELECTRIC Co., Ltd., Shizuoka, Japan), the sounds during swallowing videofluorography were recorded at a sampling frequency of 44,100 Hz with 16 bit digital resolution. Considering previous reports of swallowing-sound research, the acoustic features of swallowing sounds, and the frequency characteristics of the throat microphone, the recorded data were down-sampled to 16,000 Hz [26]. All recordings were synchronized with video recordings of swallowing videofluorography, and the body posture of each participant during ingestion was adjusted by a medical specialist to minimize the participants’ risk of aspiration. Based on a database containing 93 pieces of recording data obtained from 74 participants, including overlaps from the same participants, we worked to identify the intervals of swallowing sounds. This process involved careful labeling by a dentist based on recording data waveforms, swallowing videofluorography video, and the swallowing sounds themselves (Figure 1). The calibration for the swallowing event identification was performed by two dentists prior to labeling. Each recording datum included 1–13 swallowing-sound intervals; in total, 240 swallowing-sound intervals were recorded. 

This study was conducted with the approval (No. 3332-1) of the Tokushima University Hospital Clinical Research Ethics Review Committee.

### 2.2. Detection of Loud Events from Recorded Data

**Figure 2:**
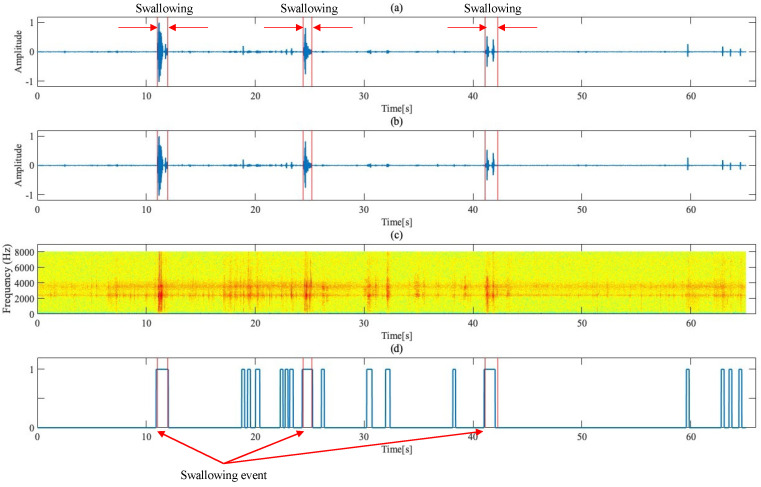
Steps from recording data to loud event detection. (**a**) Recording data waveform, including swallowing during jelly ingestion (red vertical line area is the swallowing-sound interval). (**b**) Waveform of recording data after bandpass filter processing. (**c**) Spectrogram for recording data waveform. (**d**) Recording of loud events and swallowing events (light-colored highlighted sections).

Figure 2 shows the process steps followed from recording data to detecting loud events. The frequency component of swallowing sounds is greater than 750 Hz [27], and the main component is approximately 3500 Hz [28,29]. Based on these reports, a third-order Butterworth bandpass filter with a cutoff frequency in the range of 200–8000 Hz was used, and the results of bandpass filter processing for the recording data are shown in Figure 2a,b.

Next, in this study, signals subjected to processing were framed using a frame width of 410 samples and a frameshift width of 160 samples. Subsequently, the logarithmic energy of signals in each frame was calculated (Figure 2c); then, frames exceeding the set threshold were identified as loud frames. This threshold was set through experimental trial and error as a value equal to 7.5%, which is the maximum energy value of signals. Continuous loud frames were treated as a single loud event. Furthermore, the detected loud events had an offset of 6 frames (total: 0.06 s) applied to each side and were separated by less than 0.12 s intervals. The experimentally obtained loud events were evaluated through comparison with the swallowing sound intervals that were previously defined by a dentist (Figure 2d).

In this study, based on the swallowing-sound intervals previously defined by a dentist, a labeling process was used to classify the loud events extracted from recording data into two categories: swallowing and non-swallowing. Regarding the classification criteria, if loud events overlapped swallowing-sound intervals indicated by a dentist by 35% or more, then those loud events were labeled as “swallowing sounds”. Conversely, if detected loud events overlapped the indicated swallowing-sound intervals by less than 35%, or swallowing sounds were too low in volume and not detected, they were labeled “non-swallowing sounds”. Using this method, a total of 234 swallowing-sound events and 697 non-swallowing-sound events were identified. The system followed in this study analyzes a binary classification problem (class 1: swallowing-sound events, and class 0: non-swallowing-sound events), based on these loud events.

In this study cohort comprising 49 men and 25 women (mean age: 78.01 ± 9.88 years old), we examined the results of loud event detection using the novel swallowing-sound-detection system, as well as the classification of swallowing-sound and non-swallowing-sound events.

As described earlier, a swallowing videofluorography video was recorded simultaneously with the swallowing sounds. Through a detailed analysis of the recorded data waveform and video, we identified 234 swallowing- and 697 non-swallowing-sound events. The non-swallowing-sound events were further subdivided through a manual labeling process (See Table 1).

The 697 analyzed non-swallowing-sound events included diverse sounds heard in actual clinical settings, including respiration, coughing, voice and speech, environmental sounds, and rustling of clothes (Table 1). The average duration and standard deviation for swallowing- and non-swallowing-sound events were 1.34 ± 4.19 and 0.67 ± 0.74, respectively. Notably, swallowing-sound events tended to have longer durations than non-swallowing-sound events. These non-swallowing-sound events exemplify the unique challenges encountered in clinical environments, and a database comprising such sounds was utilized to evaluate the performance of the proposed swallowing-sound and non-swallowing-sound events classification system in this study.

### 2.3. Feature Extraction

**Figure 3:**
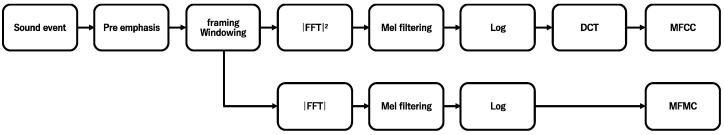
Steps for calculating the MFCC and MFMC.

In this study, we used the MFCC and MFMC as features for binary classification. As shown in Figure 3, these features were derived using different specialized steps for feature extraction from loud events.

The MFCC is a feature that is frequently used for voice recognition [30,31]. The cepstral analysis accounts for human hearing characteristics, and the MFCC has the property of aggregating features at a low order. The process in this study adopts the following procedure for feature extraction from loud events. First, a pre-emphasis filter was applied to signals to emphasize the high-frequency component. Next, we performed frame processing. Each frame had a length of 410 samples, and the shift-width between frames was set to 160 samples. A Hamming window was used in this frame processing step, and a window function was applied to each frame of signals. Thereafter, we performed 1024-point Fast Fourier Transform (FFT) on each frame and obtained a power spectrum. The converted power spectrum was multiplied by a Mel scale filter bank of 40 channels, and a logarithm was obtained for the output of each filter bank. Finally, a discrete cosine transform (DCT) was applied to these logarithm values to extract a 12-dimensional MFCC. The MFCC can effectively grasp the frequency characteristics of sound signals and particularly provide features that imitate human characteristics.

The MFMC is a technique for extracting the Mel Frequency Amplitude Coefficient from sound signals. In this study, we applied the MFMC extraction process based on reports that the MFMC is superior to the MFCC in speaker emotion recognition and early diagnosis of disease [30,32]. The MFMC extraction process followed the same procedure that was used for the MFCC in its initial stages. Specifically, we performed 1024-point FFTs on each audio frame and obtained the amplitude spectrum. Next, the obtained amplitude spectrum was multiplied by a 40-channel Mel scale filter bank to derive the logarithm for each filter-bank output. At this point, the processes were the same as for the MFCC extraction; thereafter, however, for the MFMC extraction, the DCT was not performed on the obtained logarithm amplitude values, which were instead used as direct features.

In this study, we used a method to calculate representative statistics to represent loud events based on two types of features that were extracted from frames within loud events, namely, the MFCC and MFMC. In frames included in loud events, we calculated the following basic statistics from the MFCC and MFMC: mean, standard deviation, median, range, skewness, as well as their dynamic characteristics, namely, the mean, standard deviation, median, range, and skewness of the deltas of these features. We then used these features to construct 14 feature patterns—6 each for the MFCC and MFMC and two “MIX” feature patterns comprising a combination of the MFCC and MFMC. Table 1 shows that the allocation of statistics in the MFCC feature patterns (MFCC P1–P6), MFMC feature patterns (MFMC P1–P6), and MIX feature patterns (MIX P1 and P2) differ depending on the combination of features. With these feature patterns, we performed a binary classification, and thereby quantitatively evaluated the effectiveness of features in classifying swallowing and non-swallowing in loud events.

According to the analysis of Table 2, the number of dimensions of a feature in each feature pattern tends to increase from P1 to P6. Furthermore, a similar increasing tendency is observed in MIX feature patterns, which confirm that MIX P2, in particular, has the largest number of dimensions.

### 2.4. Classification of Loud Events Using Machine Learning Models

In this proposed system, we adopted the following three machine learning models: an SVM, Multi-Layer Perceptron (MLP), and an ensemble learning model that integrates both. This was to facilitate binary classification based on the feature patterns extracted from loud events.

#### 2.4.1. Support Vector Machine

The purpose of an SVM is to determine the decision boundaries having the largest margins between two classes based on a learning data set. This achieves high identification performance for unknown data. The learning data set consists of d dimensional N of samples, $x_{\begin{array}{c} n \end{array}}\in {\mathbb{R}}^{d},1\leq n\leq N$, and a corresponding class label, starting from $y_{\begin{array}{c} n \end{array}}\in \left\{+1,-1\right\}$. In this study, we solve the following optimization problem:

(1) $$\underset{w,b,\xi_{i}\;}{\min}\frac{1}{2}w^{T}w+C\sum_{n=1}^{N}\xi_{n}$$

$$s.t.\;\;\;y_{n}\left(w^{T}x_{\begin{array}{c} n \end{array}}+b\right)-1+\xi_{n}\geq 0,\;\;\xi_{n}\geq 0$$

Here margin violations in the samples are tolerated, with w being the normal vector of the decision boundary, b the offset, and $\xi_{n}$ the slack variable. C is a regularization parameter that we used for overfitting prevention and margin balance adjustment. The optimum value of the regularization parameter, C, was determined through a grid search, and C=0.05 was selected to maximize the general purpose performance of the model.

We applied Platt scaling to convert the score obtained from this linear SVM to a posterior probability distribution, whereby the score was normalized to a range of 0 to 1 based on the distance from the separating hyperplane. Specifically, a sigmoid function was learned to output the posterior probability distribution, and this function was used for the following conversion:

(2) $$P(s_{\begin{array}{c} x \end{array}})=\frac{1}{1+\exp\left(A\;s_{\begin{array}{c} x \end{array}}+B\right)}$$

Here $s_{\begin{array}{c} x \end{array}}$ indicates the distance from the separating hyperplane, and A and B are the learned sigmoid function parameters. This method provides the output of the model in a probabilistic format that is easier to interpret.

#### 2.4.2. Multi-Layer Perceptron

The MLP is an artificial neural network that consists of three layers: an input layer, several intermediate layers, including perceptrons, and an output layer for outputting predictions in response to input vectors [33]. The input layer is fully connected to the intermediate layers, and intermediate layers to each other, and each node is connected to all nodes in the adjacent layer. For the activation function used in this study, a hyperbolic tangent sigmoid function (tanh) is used in the intermediate layers, and a SoftMax function is used in the output layer. The hyperbolic tangent function improves the expression of the model through its nonlinearity. A SoftMax function is used to express class predictive probability in the output layer. In this study, based on the results of repeated trial and error, the number of units of intermediate layers was set to the number resulting from adding 2 to the number of dimensions of a feature. We adopted a backpropagation algorithm based on the scaling conjugate grading method (SCG) for the learning process. With this algorithm, the learning process was set to stop if the number of epochs reached 2000, or when the performance evaluation gradient became smaller than a predetermined threshold.

#### 2.4.3. Ensemble Learning Model

In this study, we planned to exceed the limits of individual learners through an Ensemble Learning Machine (ELM) that combined the SVM and MLP. Specifically, we utilized the “Ensemble Learning Toolbox” [34] proposed by Riberiro et al. and used a boosting method to integrate a total of 10 learners, including five linear SVM and five MLP. Boosting is an approach involving successively training multiple learners and, by having successive learners correct the misclassification of the preceding learners, improving the overall classification performance. This process is used to grasp linear and nonlinear data characteristics and is particularly expected to enable more accurate predictions in the classification of loud events. Initially, weak learners (linear SVM or MLP) are trained using training data. Learner performance is evaluated based on its error rate and then weighted based on the results. Subsequent learners then expressly learn based on the probability distribution focused upon the samples that were misclassified by previous learners. This step is repeated until all learners have been trained. The output from each weak learner is connected using the weighting conferred. Classification as class 1 or class 0 is determined by weighted voting based on learner output. Based on the integrated output, the ensemble model determines final class labels. If the overall confidence in class 1 is higher than in class 0, it is determined to be class 1, and vice versa, it is determined to be class 0. Boosting is an approach in which overall classification performance is raised by the next learner correcting the previous learner’s errors. With this strategy, the model is expected to exhibit high adaptability and generalization ability for specific problems and provide accurate predictions, even for complex data sets and real-world problems.

### 2.5. Evaluation of Loud Event Classification with K-Fold Cross-Validation

In this study, we performed a 5-fold cross-validation to evaluate the generalization performance of the proposed system. With this method, we divided the data sets into five equal parts, used each part one at a time as a test set, and then used the remaining part as a training set. Thus, we were able to comprehensively evaluate the system’s performance.

We used the following six indices for performance evaluation: accuracy, sensitivity, specificity, positive predictive value (PPV), negative predictive value (NPV), and F1 score. These indices were calculated by the following equations based on the definition of swallowing-sound events as positive and of non-swallowing-sound events as negative:

(3) $$Accuracy\left[\%\right]=\frac{TP+TN}{TP+FP+TN+FN}\times 100$$

(4) $$Sensitivity\left[\%\right]=\frac{TP}{TP+FN}\times 100$$

(5) $$Specificity\left[\%\right]=\frac{TN}{TN+FP}\times 100$$

(6) $$PPV\left[\%\right]=\frac{TP}{TP+FP}\times 100\;$$

(7) $$NPV\left[\%\right]=\frac{TN}{FN+TN}\times 100\;$$

(8) $$F1\;Score=2\frac{Sensitivity\times PPV}{Sensitivity+PPV}$$

The percentage of correct classification for each category denotes the accuracy, whereas the percentage of correctly identified swallowing-sound events out of the total number of actual swallowing-sound events denotes sensitivity. The percentage of correctly identified non-swallowing-sound events out of the total number of non-swallowing-sound events indicates the specificity. The percentage of cases predicted to be swallowing-sound events and were actually swallowing-sound events is the PPV, whereas the NPV is the percentage of cases predicted to be non-swallowing-sound events and were actually non-swallowing-sound events. The F1 score is the harmonic mean of sensitivity and PPV and is 1 if the system has correctly classified everything. The F1 score ranges from 0 to 1 and is a particularly useful performance evaluation index if there is a bias in the number of positive and negative samples [35].

## 3. Results

### Results of Evaluating Performance of Swallowing-Sound and Non-Swallowing-Sound Event Classification System

We used a swallowing-sound and non-swallowing-sound event classification system and classified 234 swallowing-sound events and 697 non-swallowing-sound events. Furthermore, we performed a five-fold cross-validation to evaluate system classification performance. Performance evaluation was performed based on 14 feature patterns and using three machine learning models: the SVM, MLP, and ELM. The results of the evaluation of the classification performance are shown in Table 3 based on the F1 score and accuracy.

According to the results of the analysis shown in Table 3, if the feature pattern is MIX P2 and ELM is adopted as the machine learning model, an F1 score of 0.90 and an accuracy of 95.24% are achieved, and this shows the highest performance of all the evaluated combinations. MIX P2 comprehensively uses the respective basic statistics and dynamic features of the MFCC and MFMC. Here, we present the performance metrics of when the three machine learning models recorded their respectively highest F1 scores.

Table 4 shows the performance metrics (six evaluation indices, including F1 score, accuracy, sensitivity, etc.) when the three machine learning models achieved their respective highest F1 scores. The ensemble learning model has a high F1 score compared to other models and exhibits similarly high values for sensitivity and PPV, which are important for calculating the F1 score. According to the analysis of Table 3, the ELM had the highest F1 score in 11 cases out of the 14 feature patterns. Although no clear improvement was seen compared to other machine learning models with some feature patterns, these results suggest that the ELM more effectively classifies swallowing-sound events as compared to other models. Additionally, it may be broadly observed from Table 3 that classification performance tends to improve in the three machine learning models when the number of dimensions of a feature increases. Therefore, we conducted a detailed investigation of the relationship between feature patterns (number of dimensions of a feature) and classification performance (accuracy) using ensemble learning.

**Figure 4:**
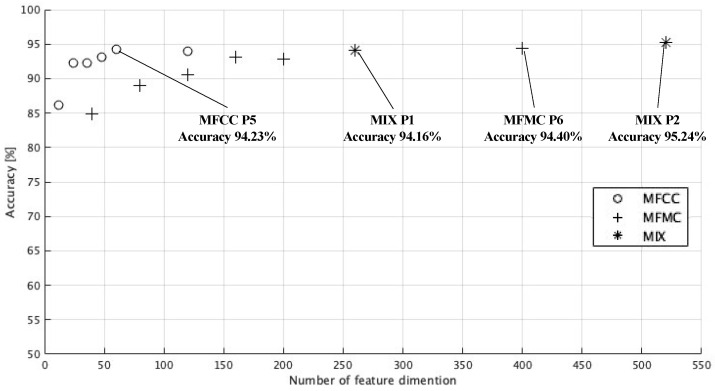
Comparison of number of feature dimensions and swallowing-sound classification performance.

Figure 4 shows a scatterplot using the classification accuracy of the proposed system (vertical axis) and the number of feature dimensions possessed by the feature patterns shown in Table 1 (horizontal axis), and represents the classification results based on the ensemble learning model. In this scatterplot, the highest classification accuracy of 95.24% is achieved with MIX P2 (number of feature dimensions 520), and the classification performance tends to improve as the number of dimensions of both the MFCC and MFMC features increases; however, there are also cases in which the classification accuracy for swallowing sounds is approximately 95%, even with relatively few numbers of feature dimensions. In particular, MFCC P5 (number of feature dimensions: 60) shows a good performance with a high classification accuracy of 94.23%, suggesting that an efficient classification may be possible, even with a small number of dimensions.

Owing to the utilization of an unbalanced data set, we re-conducted a five-fold cross-validation to classify class 1 as swallowing-sound events and class 0 as non-swallowing-sound events. Subsequently, we re-calculated the mean and standard deviation of the performance metrics for each class when identified as positive. The results are presented in Table 5. For this purpose, we utilized the feature pattern MIX P2 and adopted the ELM as the machine learning model.

Our analysis indicates minimal differences among classes, suggesting that our method yields a high detection performance for swallowing sounds. The F1-macro average, computed by averaging the mean F1 scores for class 1 and class 0, is approximately 0.92. These outcomes demonstrate the effectiveness of the feature pattern and machine learning model used, highlighting their suitability for handling unbalanced data sets in our study.

## 4. Discussion

In this study, we undertook an automatic classification of swallowing-sound and non-swallowing-sound events, for which we built three machine learning models and compared their performance. Based on the results, if we exclude the feature patterns for MFCC P4, MFMC P2, and MFMC P5, the ELM exhibited the highest classification performance. 

Zhao et al. [33] used vibration signals from the pharynx obtained from healthy persons and patients with dysphagia, and, with an integrated classifier combining Adaboost, an SVM, and MLP, reported 72.09% accuracy for detecting dysphagia. Similarly, these results suggest that an ELM would effectively detect swallowing sounds from specific clinical recording data. A model including MFCC features, in particular, demonstrated good performance. The MFCC is a feature that is widely used for voice recognition [30,31] and for identifying swallowing sounds [23,36]. In this study, feature patterns using the MFCC as the basis showed the most superior performance. Furthermore, we adopted MFMC features that are reportedly effective for speech emotion recognition; however, no significant difference in performance was seen as compared to the analysis using MFCC feature patterns. This may be due to differences in the type and scale of the databases used.

Table 6 summarizes a literature review of the past 15 years, with the latest study results on the automated detection of swallowing sounds. Sazonov et al. used the Mel Scale Fourier Spectrum and reported approximately 85% accuracy in classifying swallowing sounds using an SVM, with data sets including patients who were obese [37]. Moreover, Khlaifi et al. used a classification method that calculates a single MFCC as an acoustic feature for automatically detected acoustic events and, with a GMM and EM algorithm, it was possible to classify swallowing at a recognition rate of 95.94% from acoustic events obtained from a swallowing data set of only healthy persons [23]; the authors used features that mimic human hearing characteristics. In this study, we used basic statistics of the MFCC and feature patterns including dynamic features, and confirmed that classification performance clearly improves in actual clinical recording data more than when these feature patterns use a single MFCC. In addition, in a study of the construction of a swallowing-sound-detection system using a recording database of patients with dysphagia and healthy participants, the real-time detection system for swallowing sounds that was proposed by Jayatilake et al. detected loud intervals and identified swallowing sounds based on their duration and frequency. Swallowing sounds recorded in repetitive saliva swallowing tests performed on healthy participants were reportedly detectable with 83.7% precision and 93.9% recall when using a proposed algorithm. Furthermore, the results show that swallowing sounds when patients with dysphagia swallow 3 mL of water (25% barium mixture) can be detected with 79.3% accuracy [38]. Kuramoto et al. reported that it was possible to classify swallowing sounds and noise with 97.3% accuracy with a Convolutional Neural Network (CNN) model using spectrogram features [24]. However, a direct comparison is difficult due to a lack of evaluation of other performance metrics. Nevertheless, it is noteworthy that with the technique reported here, we were able to achieve a performance comparable to theirs even when using recording data from patients with dysphagia that contained more complex swallowing-sound patterns than those in healthy individuals, and when using shallow learning [39,40].

Swallowing detection was previously possible with various sensors, such as microphones, respiratory flow, and electromyograms [25,36,41,42,43]. In recent years, studies of swallowing-sound detection have combined high-resolution CA (HRCA) signals and deep learning. Khalifa et al. reported that spectrograms of signals from 248 patients with dysphagia that were obtained from three accelerometers and microphones, and learning using a DNN, enabled the detection of swallowing with accuracy greater than 95% [25]. However, if multiple sensors are used, and they are difficult to attach, the necessity of making adjustments to attachment positions per patient and the burden on patients are considered, then these methods are associated with issues that hamper their practical use in clinical settings [44]. In this study, we adopted an approach that involved using microphones attached only around the pharynx to detect swallowing, which has significant advantages in simplifying attachment and mitigating the patient burden.

The method for automatically detecting swallowing sounds using actual clinical condition data in this study achieved high detection accuracy under specific conditions, albeit with several constraints. Although a very small number of errors occurred. The main causes of these errors are identified as a decreased signal-to-noise ratio (SNR) in swallowing sounds and sound episodes with multiple voices mixed together. To resolve these issues, it will be necessary to expand the database and develop more advanced feature extraction methods. 

The method may occasionally detect long swallowing-sound events. Future analyses may require the implementation of techniques for emphasizing swallowing sounds.

The scale of data sets used was small, and there was limited diversity among the patients; therefore, the possibility of generalizing this method requires further validation. Moreover, additional testing will be essential for the evaluation of its robustness to interference that may be encountered in actual medical settings, such as external noise and patient motions. It will be important to take these constraints into account in future studies to focus on diverse validations using wider data sets, and thereby test robustness in actual clinical environments.

## 5. Conclusions

In this study, we proposed a novel method for automatically detecting swallowing sounds using recording data obtained in actual clinical settings from 74 patients with dysphagia. By extracting basic statistics and dynamic features based on the MFCC and MFMC, and adopting an ensemble learning model, we achieved superior performance with an F1-macro average of approximately 0.92 and an accuracy of 95.20%. The method developed in this study has demonstrated efficient applicability to swallowing-sound detection in the current clinical recording database. Therefore, we believe that the proposed method will contribute to enhancing objectivity in CA.

## Figures and Tables

**Table 1:** Results of the manual labeling-based classification of loud events.

Sound Event	Total
Swallowing	234
Respiration	41
Cough	38
Voice·Speech	224
Environmental sounds, rustling sound	394
Total	931

**Table 2:** Feature patterns and number of dimensions. ○ represents patterns of MFCC, while ● represents patterns of MFMC.

	Mean	Standard Deviation	Median	Range	Skewness	Dynamic Features	Number of Feature Dimension
MFCC P1	○						12
MFCC P2	○	○					24
MFCC P3	○	○	○				36
MFCC P4	○	○	○	○			48
MFCC P5	○	○	○	○	○		60
MFCC P6	○	○	○	○	○	○	120
MIMC P1	●						40
MFMC P2	●	●					80
MFMC P3	●	●	●				120
MFMC P4	●	●	●	●			160
MFMC P5	●	●	●	●	●		200
MFMC P6	●	●	●	●	●	●	400
MIX P1	○●	○●	○●	○●	○●		260
MIX P2	○●	○●	○●	○●	○●	○●	520

**Table 3:** Results of 5-fold cross-validation based on 14 feature patterns and using three types of machine learning models.

Feature Pattern	SVM		MLP		ELM	
	F1 Score	Accuracy [%]	F1 Score	Accuracy [%]	F1 Score	Accuracy [%]
MFCC P1	0.57	77.74	0.71	84.61	0.73 ^h^	86.10
MFCC P2	0.84 ^h^	91.98	0.82	90.82	0.84 ^h^	92.29
MFCC P3	0.85 ^h^	92.20	0.83	91.15	0.85 ^h^	92.27
MFCC P4	0.87	93.79	0.88 ^h^	93.56	0.87	93.16
MFCC P5	0.88	93.93	0.88	94.16	0.89 ^h^	94.23
MFCC P6	0.87	93.39	0.88 ^h^	94.17	0.88 ^h^	93.91
MFMC P1	0.55	79.64	0.67	84.11	0.71 ^h^	84.88
MFMC P2	0.78	88.74	0.80 ^h^	89.00	0.79	88.96
MFMC P3	0.79	89.43	0.81	90.23	0.83 ^h^	90.61
MFMC P4	0.84	91.79	0.84	91.45	0.86 ^h^	93.04
MFMC P5	0.87 ^h^	93.14	0.85	91.75	0.86	92.89
MFMC P6	0.87	93.48	0.89 ^h^	94.66	0.89 ^h^	94.40
MIX P1	0.87	93.62	0.89 ^h^	94.43	0.89 ^h^	94.16
MIX P2	0.88	93.88	0.89	94.77	0.90 ^h^	95.24

“^h^”: Highest F1 score of the three models.

**Table 4:** Feature patterns and performance evaluation values when the classification performance was highest for each machine learning model.

Machine Learning Model	Feature Pattern	F1 Score	Accuracy [%]	Sensitivity [%]	Specificity [%]	PPV [%]	NPV [%]
SVM	MFCC P5 MIX P2	0.88	93.93	86.65	96.26	88.78	95.43
		0.88	93.88	87.80	96.21	88.09	95.57
MLP	MFMC P6	0.89	94.66	88.89	96.64	90.09	96.22
ELM	MIX P2	0.90	95.24	90.14	96.88	91.09	96.83

**Table 5:** Average and standard deviation of performance metrics for positive identification of swallowing-sound event (class 1) vs. non-swallowing-sound event (class 0).

Performance Metrics	Class 0	Class 0	Class 1	Class 1
	Mean	SD	Mean	SD
Accuracy [%]	95.2	0.7	95.2	0.7
Sensitivity [%]	93.74	4.67	91.66	4.34
Specificity [%]	94.24	3.77	96.31	1.96
F1 score	0.92	0.04	0.91	0.02
PPV [%]	89.95	5.35	89.68	4.96
NPV [%]	96.94	1.52	97.21	1.13

SD: standard deviation.

**Table 6:** Comparison with other studies on swallowing-sound detection,.

References	Data Sets	Feature Sets	Method	Result
Sazonov et al. (2010) [37]	13 healthy subjects 7 obese subjects	Wavelet Packet Decomposition (WPD) Mel Scale Fourier Spectrum (msFS)	Machine learning (SVM)	Average weighted accuracy 84.7%
Jayatilake et al. (2015) [38]	70 subjects with dysphagia 15 healthy subjects	―	Based on wavelet transform and zero crossing rate	Accuracy Water swallowing: 79.3% Dry or salvia swallowing: 83.7%
Khlaifi et al. (2018) [23]	14 healthy subjects	Mel Frequency Cepstral Coefficient (MFCC)	Machine learning (GMM)	Recognition rate UTC database: 84.57% Grenoble database: 95.94%
Kuramoto et al. (2020) [24]	Healthy subjects and subjects with dysphagia: in total, 226 subject	Spectrogram from swallowing sound	Machine learning (CNN)	Accuracy: 97.3%
Ours	74 subjects with dysphagia	Mel Frequency Cepstrum Coefficient (MFCC) Mel Frequency Magnitude Coefficient (MFMC)	Machine learning (ensemble learning model)	F1-micro average: approximately 0.92 Accuracy: 95.20%

## Data Availability

Data are unavailable due to privacy or ethical restrictions.
